# Significance of Platinum‐Based Chemotherapy With Programmed Death‐1 Blockade in Limited Disease Small Cell Lung Cancer: A Retrospective Study

**DOI:** 10.1111/1759-7714.70118

**Published:** 2025-06-30

**Authors:** Ayako Shiono, Hisao Imai, Kyoichi Kaira, Takanori Abe, Yuki Sato, Ken Yamamoto, Hiroki Watanabe, Yuko Tsuchiya‐Kawano, Akihiro Tamiya, Takashi Osaki, Noriko Yanagitani, Shigeru Tanzawa, Toshiyuki Sumi, Kohei Yoshimine, Yohei Matsui, Satoshi Endo, Kazuhiko Shibata, Shinnosuke Takemoto, Yosuke Miura, Yoshiaki Nagai, Junichi Nakagawa, Takeshi Tsuda, Hiroshi Kagamu

**Affiliations:** ^1^ Department of Respiratory Medicine Saitama Medical University International Medical Center Hidaka Saitama Japan; ^2^ Division of Respiratory Medicine Gunma Prefectural Cancer Center Ota Gunma Japan; ^3^ Department of Radiation Oncology Saitama Medical University International Medical Center Hidaka Saitama Japan; ^4^ Department of Respiratory Medicine Kobe City Medical Center General Hospital Kobe Hyogo Japan; ^5^ Division of Thoracic Oncology Kobe Minimally Invasive Cancer Center Kobe Hyogo Japan; ^6^ Department of Internal Medicine Niigata Cancer Center Hospital Niigata Niigata Japan; ^7^ Department of Respiratory Medicine Kitakyushu Municipal Medical Center Kitakyushu Fukuoka Japan; ^8^ Department of Internal Medicine NHO Kinki‐Chuo Chest Medical Center Sakai Osaka Japan; ^9^ Department of Respiratory Medicine NHO Shibukawa Medical Center Shibukawa Gunma Japan; ^10^ Department of Thoracic Medical Oncology The Cancer Institute Hospital of Japanese Foundation for Cancer Research Koto‐ku Tokyo Japan; ^11^ Division of Medical Oncology, Department of Internal Medicine Teikyo University School of Medicine Itabashi‐ku Tokyo Japan; ^12^ Department of Pulmonary Medicine Hakodate Goryoukaku Hospital Hakodate Hokkaido Japan; ^13^ Department of Respiratory Medicine Iizuka Hospital Iizuka Fukuoka Japan; ^14^ Department of Pulmonary Medicine Kyoto Prefectural University of Medicine Kyoto Kyoto Japan; ^15^ Division of Medical Oncology, Department of Medicine Kouseiren Takaoka Hospital Takaoka Toyama Japan; ^16^ Department of Respiratory Medicine Nagasaki University Graduate School of Biomedical Sciences Nagasaki Nagasaki Japan; ^17^ Department of Respiratory Medicine Gunma University Graduate School of Medicine Maebashi Gunma Japan; ^18^ Department of Respiratory Medicine Jichi Medical University, Saitama Medical Center Saitama Saitama Japan; ^19^ Department of Respiratory Medicine NHO Takasaki General Medical Center Takasaki Gunma Japan; ^20^ Division of Respiratory Medicine Toyama Prefectural Central Hospital Toyama Toyama Japan

**Keywords:** chemoradiotherapy, limited disease small cell lung cancer, PD‐1 blockade, recurrence, retrospective

## Abstract

**Main Problem:**

The efficacy and safety of platinum‐based chemotherapy with programmed death‐1 (PD‐1) blockade after chemoradiotherapy (CRT) for the treatment of limited disease (LD) small cell lung cancer (SCLC) is unknown. This study aimed to assess the effectiveness and tolerability of platinum‐based chemotherapy with PD‐1 blockade in patients with recurrent LD‐SCLC after CRT.

**Methods:**

This retrospective study analyzed 66 patients who experienced recurrence after CRT for LD‐SCLC and received platinum‐based chemotherapy with PD‐1 blockade therapy between August 2019 and September 2020 at 19 Japanese institutions. Clinical efficacy was assessed according to response rate, survival, and toxicity.

**Results:**

The overall response rate was 53.0% (95% confidence interval [CI], 48.9–65.0), and the disease control rate was 78.7% (95% CI, 68.9–88.5). The median progression‐free survival and overall survival periods were 5.9 (95% CI, 4.7–7.3) months and 24.9 (95% CI, 16.8–28.1) months, respectively. The frequencies of grade ≥ 3 hematological adverse events were as follows: leukopenia, 47.0%; neutropenia, 65.2%; and febrile neutropenia, 8.3%. There was no treatment‐related death.

**Conclusions:**

Chemoimmunotherapy is a feasible and effective treatment for recurrent disease after CRT in patients with LD‐SCLC, providing a new potential option for the pharmacological management of these patients.

## Graphical abstract

The median progression‐free survival and overall survival periods were 5.9 (95% CI, 4.7–7.3) and 24.9 (95% CI, 16.8–28.1) months, respectively. Chemoimmunotherapy is an effective treatment for recurrent disease after chemoradiotherapy in patients with limited disease small cell lung cancer.

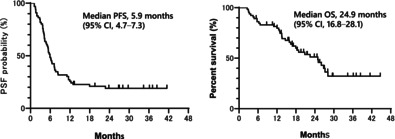

## 1 Introduction

Small cell lung cancer (SCLC) accounts for approximately 10%–15% of all lung cancers [1]. SCLC is a highly malignant tumor that grows rapidly and can cause lymph node and distant metastases at an early stage; however, it is characterized by its high sensitivity to radiotherapy and chemotherapy. Patients who receive initial chemotherapy and chest radiotherapy for limited disease (LD)‐SCLC show a good response, but the 5‐year survival rate is < 30% [2]. The standard treatment for LD‐SCLC is a combination of chemotherapy and thoracic radiotherapy, followed by prophylactic cranial irradiation (PCI) for patients who respond to chemoradiotherapy (CRT) [3, 4]. Two meta‐analyses showed that the combination of chemotherapy and thoracic radiotherapy for LD‐SCLC improved the overall survival (OS) compared with chemotherapy alone [5, 6]. According to previous studies, the prognosis of patients with LD‐SCLC receiving CRT is 24–30 months [7, 8, 9]. Furthermore, the effects of conventional treatments on recurrent SCLC are limited. The OS periods for recurrent SCLC are 5.9–8.0 months with nogitecan [10, 11, 12], 8.1–9.2 months with amrubicin [13, 14], and 7.5 months with carboplatin + etoposide (retreatment) [15]. These trials included relapses after CRT in patients with LD‐SCLC. Unfortunately, none of these treatments have resulted in long‐term survival.

Platinum‐based chemotherapy with programmed death‐1 (PD‐1) blockade has been used in clinical practice to treat extensive disease (ED)‐SCLC. In the phase III trials IMpower133 [16] and CASPIAN [17], the addition of atezolizumab or durvalumab to a platinum and etoposide regimen significantly improved the OS of patients who had not received prior treatment (in IMpower 133, 12.3 months vs. 10.3 months; in CASPIAN, 13.0 months vs. 10.3 months). Although there was a tendency for an increase in immune‐related adverse events (irAEs), such as skin rash, infusion reaction, and abnormal thyroid function in clinical trials, there was no increase in grade 3 or higher toxicity. The efficacy of platinum‐based chemotherapy with PD‐1 blockade for SCLC that relapses after systemic chemotherapy remains unclear. However, some studies have suggested that combination therapy may be an effective treatment option. Ishii et al. [18] investigated the efficacy of platinum‐based chemotherapy with PD‐1 blockade in patients with SCLC with a history of platinum‐based chemotherapy. The overall response rate (ORR), disease control rate (DCR), and median progression‐free survival (PFS) period were 37.5%, 75.0%, and 4.0 months, respectively. Shang et al. [19] reported that patients who received an immune checkpoint inhibitor (ICI) rechallenge had longer OS than did those without rechallenge. In patients with fewer than four metastatic sites, there was a significant difference in OS between the ICI retreatment and non‐ICI rechallenge groups (*p* = 0.036). Patients harboring fewer than four metastatic sites had fewer M2 macrophages and more CD4 naïve T‐cell infiltration. These findings suggest that this regimen may be applicable to recurrent LD‐SCLC, which is expected to have a relatively small tumor volume compared with that in ED‐SCLC. However, there are no reports on the efficacy and safety of platinum‐based chemotherapy with PD‐1 blockade in patients with LD‐SCLC who experience recurrence after CRT. To the best of our knowledge, this is the first such report to date. Therefore, this retrospective study aimed to evaluate the efficacy and safety of platinum‐based chemotherapy with PD‐1 blockade after CRT for LD‐SCLC.

## 2 Methods

### 2.1 Study Patients

This retrospective study analyzed patients who experienced recurrence after CRT for LD‐SCLC and received platinum‐based chemotherapy with PD‐1 blockade therapy between August 2019 and September 2020 at 19 Japanese institutions. The following patients were included in this study: (1) patients with SCLC diagnosed based on cytology or histology, (2) patients with recurrence after CRT as initial treatment for LD‐SCLC, and (3) patients with recurrent disease after CRT who were treated with platinum and etoposide plus atezolizumab or durvalumab as first‐line combination chemotherapy for LD‐SCLC. LD‐SCLC was defined as a disease limited to the ipsilateral thorax, contralateral mediastinum, and contralateral supraclavicular fossa lymph nodes without malignant pleural or pericardial effusion.

All patients underwent chest radiography, thoracoabdominal computed tomography, brain computed tomography or magnetic resonance imaging, and [18] F‐fluorodeoxyglucose positron emission tomography or bone scintigraphy before treatment to assess the tumor‐node‐metastasis stage. Patient information was collected from electronic medical records and analyzed to identify the eligible patients.

The Institutional Review Board of the International Medical Center, Saitama Medical University, approved the study protocol (number 2023‐089). This study followed the institutional and national ethical standards and complied with the Declaration of Helsinki (2013 revision). The need for informed consent was waived by the institutional review boards of the participating institutions because of the retrospective nature of the study. However, an opportunity to refuse participation was provided using the opt‐out method.

### 2.2 Treatment and Response Evaluation

All the patients received concurrent or sequential radiotherapy and platinum plus etoposide chemotherapy after being diagnosed with LD‐SCLC. Each patient received platinum (cisplatin or carboplatin) and etoposide at doses determined by the attending physician, in accordance with product labeling. Three‐dimensional conformal or intensity‐modulated radiotherapy was used depending on the machine resources of the facility. The total doses were 45 Gy in 30 fractions (1.5 Gy twice daily) for the accelerated hyperfractionated regimen and 60 Gy in 30 fractions (2 Gy once daily) for the sequential radiotherapy regimen. Elective nodal irradiation was performed; however, in some cases, field radiotherapy was used to reduce the dose to normal organs. After definitive radiotherapy, PCI was performed for patients who responded well to the initial treatment. The total dose was 25 Gy delivered in 10 PCI fractions. PCI is usually performed within 2 months of the end of CRT.

The patients received platinum‐based chemotherapy with PD‐1 blockade after recurrence. Each patient received up to four cycles of atezolizumab (fixed dose of 1200 mg, intravenous injection on Day 1 of each cycle), carboplatin (area under the curve of 4–5 min mg/mL, intravenous injection on Day 1 of each cycle), and etoposide (80–100 mg/m^2^ body surface area, intravenous injection on Days 1–3 of each cycle), followed by atezolizumab maintenance every 3 weeks, or durvalumab (fixed dose of 1500 mg, intravenous injection on Day 1 of each cycle), platinum (carboplatin [area under the curve of 4–5 min mg/mL, intravenous injection on Day 1 of each cycle]) or cisplatin (60–80 mg/m^2^ body surface area, intravenous injection on Day 1 of each cycle), and etoposide (80–100 mg/m^2^ body surface area, intravenous injection on Days 1–3 of each cycle), followed by durvalumab maintenance every 4 weeks. The appropriateness of dose reduction and the use of granulocyte colony‐stimulating factor (G‐CSF) as prophylaxis against neutropenia preparations were determined by a physician. If the treatment failed, the patient was referred for subsequent treatment, which included the best supportive care.

Radiographic treatment responses were evaluated according to the best overall treatment response and maximum tumor shrinkage based on the Response Evaluation Criteria in Solid Tumors version 1.1 [20]. Tumor response was classified as complete response (CR), partial response (PR), stable disease (SD), progressive disease (PD), and not evaluated (NE). Treatment toxicities associated with combination chemotherapy were graded according to the Common Terminology Criteria for Adverse Events (version 5.0).

At treatment initiation, the body mass index (BMI) was calculated by dividing the body weight (kg) by the square of the height (m). We investigated the potential correlation between BMI and the effectiveness of platinum‐based chemotherapy with PD‐1 blockade using a BMI cutoff value of 22.0 kg/m^2^, which represents the ideal BMI for the Japanese population [21] (high BMI, ≥ 22.0 kg/m^2^; low BMI, < 22.0 kg/m^2^). We also investigated the correlation between neuroendocrine tumor markers, namely pro‐gastrin‐releasing peptide and neuron‐specific enolase, and chemoimmunotherapy using the upper limit of the normal range as the cutoff values for the tumor markers. The neutrophil‐to‐lymphocyte ratio (NLR) was calculated as the ratio of the absolute neutrophil count to the absolute lymphocyte count (threshold value: ≥ 5 to < 5) [22, 23], and a cutoff NLR of 5.0 was used to identify low‐risk (< 5.0) and high‐risk (≥ 5.0) patients. For the platelet‐to‐lymphocyte ratio (PLR), which is defined as the ratio of the absolute platelet count to the absolute lymphocyte count, a cutoff value of 185 [24] was used to identify low‐risk (< 185) and high‐risk (≥ 185) patients. The advanced lung cancer inflammation index (ALI) combines BMI, albumin, and NLR ([BMI × albumin]/NLR). We used an established cutoff score of 24 to categorize patients into low ALI (< 24) and high ALI (≥ 24) groups [25]. The prognostic nutritional index (PNI) is calculated using the serum albumin concentration and absolute lymphocyte count as follows: PNI = (10 × albumin [g/dL]) + (0.005 × absolute lymphocyte count [per mm^3^]). We used an established cutoff score of 45 to stratify patients into the low PNI (< 45) and high PNI (≥ 45) groups [26, 27, 28]. These groups were used in univariable and multivariable analyses of PFS and OS.

### 2.3 Statistical Analyses

PFS was defined as the interval from platinum‐based chemotherapy with PD‐1 blockade to disease progression. Patients who did not die within the observation period were censored on the date of their last visit or follow‐up. OS was defined as the interval from platinum‐based chemotherapy with PD‐1 blockade initiation to death or censoring on the date of the last follow‐up. Categorical variables were analyzed using Fisher's exact test, and continuous variables were analyzed using Welch's *t*‐test. The Kaplan–Meier method was used to estimate median survival times. In addition, Cox regression analysis was used to investigate the possibility that the prognosis may be affected by other factors. The significance level for the test was 0.05, and the confidence coefficient for calculating the confidence interval (CI) was 95%. As the analysis in this study was exploratory, no adjustment for multiple tests was performed. All statistical analyses were performed using the JMP statistical software, version 17.0, for Windows (SAS Institute, Cary, NC, USA).

## 3 Results

### 3.1 Patient Characteristics

Table 1 shows patient characteristics. Sixty‐six patients received platinum‐based chemotherapy with PD‐1 blockade (atezolizumab or durvalumab) as first‐line treatment after disease progression following CRT in our study, and the median duration from the start of CRT to recurrence was 9.4 (range, 3.8–91.3) months. In total, 27 patients (40.9%) had brain metastases, 10 (15.2%) had liver metastases, and 6 (9.1%) had bone metastases. Of the 27 patients with brain metastases, 6 received PCI and 11 received radiation therapy before chemoimmunotherapy. The median number of platinum‐based chemotherapies with PD‐1 blockade was 4 (range, 1–6) cycles, and dose reductions of etoposide or both etoposide and platinum were performed in 27 (40.9%) patients. Atezolizumab was frequently administered to 46 (69.7%) patients for PD‐1 blockade. The median number of PD‐1 blockade maintenance administrations was 2 (range, 0–53) cycles. Thirty‐seven (56.1%) patients received prophylactic G‐CSF treatment. The median duration of the observation period was 16.8 (range, 1.9–44.7) months. Table S1 shows the patient characteristics at the time of CRT.

**TABLE 1:** Baseline characteristics of patients.

	Total patients (*N* = 66)	%
Sex		
Male	55	83.3
Female	11	16.7
Age (years)		
Median	70	
Range	54–83	
ECOG‐PS		
0	26	39.4
1	36	54.5
2	3	4.5
3	1	1.5
Smoking status		
Yes	64	97.0
No	2	3.0
Histology		
Small cell carcinoma	64	97.0
Combined small cell carcinoma	2	3.0
History of operation		
Yes	3	4.5
No	63	95.5
History of postoperative adjuvant chemotherapy		
Yes	0	0.0
No	66	100.0
Duration from the start of chemoradiotherapy to recurrence (months)		
Median	9.4	
Range	3.8–91.3	
Presence of local recurrence at recurrence		
Yes	26	39.4
No	40	60.6
Presence of distant metastasis at recurrence		
Yes	44	66.7
No	22	33.3
Intracranial metastases at recurrence		
Yes	27	40.9
No	39	59.1
Liver metastases at recurrence		
Yes	10	15.2
No	56	84.8
Bone metastases at recurrence		
Yes	6	9.1
No	60	90.9
Chemotherapy regimen		
CBDCA + etoposide + atezolizumab	46	69.7
CDDP + etoposide + durvalumab	4	6.1
CBDCA + etoposide + durvalumab	16	24.2
ECOG‐PS for each chemotherapy regimen		
CBDCA + etoposide + atezolizumab		
0	18	27.3
1	25	37.9
2	2	3.0
3	1	1.5
CDDP + etoposide + durvalumab		
0	2	3.0
1	2	3.0
2	0	0.0
3	0	0.0
CBDCA + etoposide + durvalumab		
0	6	9.1
1	9	13.6
2	1	1.5
3	0	0.0
Number of cycles of chemotherapy administered		
Median	4	
Range	1–6	
Chemotherapy dose reduction		
Yes	27	40.9
No	39	59.1
Reason for dose reduction of chemotherapy administration		
Adverse events	26	39.4
Others	1	1.5
Reason for discontinuation of chemotherapy administration		
Progressive disease	6	9.1
Adverse events	4	6.1
Deterioration of PS	1	1.5
Others	3	4.5
Number of cycles of ICI maintenance		
Median	2	
Range	0–53	
With or without G‐CSF prophylaxis		
Yes	37	56.1
No	29	43.9
Alive at date cutoff		
Alive	32	48.5
Death	34	51.5

Abbreviations: CBDCA, carboplatin; CDDP, cisplatin; CRT, chemoradiotherapy; ECOG‐PS, Eastern Cooperative Oncology Group Performance Status; G‐CSF, granulocyte colony‐stimulating factor; PtE + ICI, platinum + etoposide + immune checkpoint inhibitor.

### 3.2 Treatment Response and Survival

**FIGURE 1:**
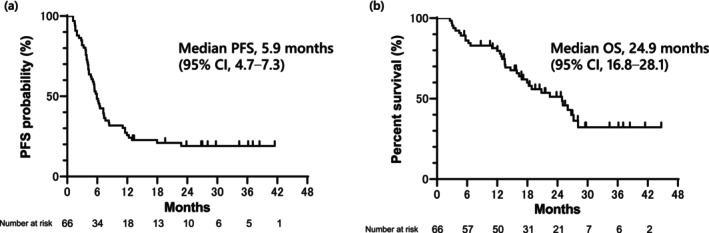
Kaplan–Meier curves for survival. (a) PFS of patients with small cell lung cancer who received platinum‐based chemotherapy with PD‐1 blockade in recurrent LD‐SCLC (median, 5.9 months). (b) OS of patients with small cell lung cancer who received platinum‐based chemotherapy with PD‐1 blockade in recurrent LD‐SCLC (median, 24.9 months). CI, confidence interval; LD‐SCLC, limited disease small cell lung cancer; OS, overall survival; PD‐1, programmed death‐1; PFS, progression‐free survival.

In this study, the treatment responses were CR in 2 patients, PR in 33 patients, SD in 17 patients, PD in 12 patients, and NE in 2 patients (Table 2). The ORR was 53.0% (95% CI, 48.9–65.0), and the DCR was 78.7% (95% CI, 68.9–88.5). The median PFS and OS periods were 5.9 (95% CI, 4.7–7.3) months and 24.9 (95% CI, 16.8–28.1) months, respectively (Figure 1a,b). Fifty‐three (80.3%) patients experienced disease progression, and 34 (51.5%) patients died during the observation period.

**TABLE 2:** Treatment response.

	Total (*N* = 66)
Response	
Complete response	2
Partial response	33
Stable disease	17
Progressive disease	12
Not evaluated	2
Response rate (%) (95% CI)	53.0 (40.9–65.0)
Disease control rate (%) (95% CI)	78.7 (68.9–88.5)

Abbreviation: CI, confidence interval.

Multivariable analysis (Table S2) identified recurrence at 12 months after CRT initiation and the presence of liver metastases at recurrence as statistically significant factors associated with PFS (*p* < 0.05). Recurrence at 12 months after CRT initiation, presence of intracranial metastases at recurrence, presence of liver metastases at recurrence, and irradiation using three‐dimensional (3D)‐CRT were statistically significant factors associated with OS (*p* < 0.05). The median PFS period was 5.2 (95% CI, 4.10–6.24) months for patients who relapsed within 12 months of starting CRT. However, the median PFS period was 9.5 (5.3–22.7) months (log‐rank *p* < 0.05) for patients who experienced recurrence at ≥ 12 months. The median OS period was 16.8 (95% CI, 12.9–24.9) months for patients who relapsed within 12 months of starting CRT. The median OS was not reached (95% CI, 26.0–not reached) for patients who experienced recurrence ≥ 12 months after the start of CRT (log‐rank *p* < 0.05).

### 3.3 Toxicity

One or more AEs occurred in 65 of the total number of patients. Table 3 presents the AEs. The most common AE observed was myelosuppression. Thirty‐one (47%) patients presented with a grades 3–4 white blood cell decrease, and 43 (65.2%) patients presented with grades 3–4 neutropenia. Febrile neutropenia was observed in 10 (10.6%) patients. IrAEs were less common than chemotherapy‐induced events caused by chemotherapy. The grades 3–4 irAEs were pneumonitis, skin rash, arthralgia, and cytokine release syndrome in one patient each (1.5%). Four patients experienced AEs that necessitated discontinuation of treatment: carboplatin allergy, colitis, pneumonitis (as an irAE), and pneumonia caused by coronavirus disease 2019. None of the patients experienced treatment‐related deaths.

**TABLE 3:** Adverse events.

Adverse events	All patients (*N* = 66)			
	Any grade	%	Grade ≥ 3	%
Led to discontinuation	3	4.5	1	1.5
Led to death	0	0.0	0	0.0
Treatment‐related^a^				
White blood cell decrease	46	69.7	31	47.0
Neutrophil count decrease	48	72.7	43	65.2
Anemia	45	68.2	9	13.6
Platelet count decrease	49	74.2	20	30.3
Febrile neutropenia	7	10.6	7	10.6
Fatigue	23	34.8	3	4.5
Anorexia	16	24.2	2	3.0
Vomiting	5	7.6	1	1.5
Diarrhea	4	6.1	2	3.0
Dyspnea	5	7.6	1	1.5
Aspartate aminotransferase increase	11	16.7	1	1.5
Alanine aminotransferase increase	11	16.7	2	3.0
Infection	5	7.6	3	4.5
Immune‐related^b^				
Pneumonitis	5	7.6	1	1.5
Skin rash	9	13.6	1	1.5
Hypothyroidism	8	12.1	0	0.0
Adrenal insufficiency	2	3.0	0	0.0
Arthralgia	3	4.5	1	1.5
Colitis	1	1.5	0	0.0
Myocarditis	1	1.5	0	0.0
Cytokine release syndrome	1	1.5	1	1.5

^a^ Treatment‐related adverse events of grade ≥ 3 in ≥ 1 patient.

^b^ Immune‐related adverse events in ≥ 1 patient.

### 3.4 Subsequent Treatments

Table 4 shows the details of the treatments administered after disease progression. Of the 51 patients who experienced relapse after platinum‐based chemotherapy with a PD‐1 blockade, 37 received subsequent treatments. The most common second‐line treatment was amrubicin monotherapy, and the most common third‐ or subsequent‐line treatment was irinotecan monotherapy. Fourteen patients were treated with the best supportive care.

**TABLE 4:** Subsequent treatment.

	Second‐line	Third‐line	Fourth‐line or beyond
Carboplatin + etoposide	4	1	0
Carboplatin + etoposide + atezolizumab/durvalumab	0	0	0
Carboplatin + irinotecan	1	0	0
Carboplatin + paclitaxel	0	1	3
Amurubicin	33	6	0
Nogitecan	0	1	1
Irinotecan	2	11	0
Others	0	2	2
Best supportive care	14	—	—

## 4 Discussion

In this study, the efficacy and safety of platinum‐based chemotherapy with PD‐1 blockade were evaluated after CRT in patients with recurrent LD‐SCLC. The ORR was 53.0%, and the PFS and OS periods were 5.9 and 24.9 months, respectively; these indicated a better prognosis than that with conventional treatment after recurrence of CRT [10, 11, 12, 13, 14, 15], IMpower133 [16], and CASPIAN [17]. Moreover, the profile of AEs was similar to that with conventional treatments and has been considered tolerable [10, 11, 12, 13, 14, 15] in clinical trials. To the best of our knowledge, this is the first study to evaluate the efficacy and safety of platinum‐based chemotherapy with PD‐1 blockade therapy in patients with recurrent LD‐SCLC after CRT.

In this study, the OS period from the start of CRT was 34.6 months (95% CI, 28.3–74.8, Figure S1); this was a better prognosis than that in the era before the addition of ICI to SCLC, and the impact of ICI on prognosis may be significant. However, because no control group was established in this study, it is difficult to determine whether the improvement in OS is truly attributable to immunotherapy. It is possible that patients with relatively good systemic conditions who received ICI treatment contributed to the results. As LD‐SCLC is generally monitored regularly after CRT, the tumor volume may be smaller than that in patients with advanced malignancies, and this may have contributed to the trend toward longer OS. In this study, recurrence 12 months after CRT initiation, the presence of liver metastasis, brain metastasis, and 3D‐CRT were independent prognostic factors associated with OS (Table S2). The presence of brain or liver metastases is an independent poor prognostic factor for SCLC [29, 30, 31, 32, 33]. There is a lack of consensus among meta‐analyses regarding the efficacy of ICIs in the treatment of ED‐SCLC with liver metastases. One meta‐analysis of ED‐SCLC with liver metastases demonstrated that the addition of ICIs to chemotherapy resulted in a significantly improved OS compared with that after chemotherapy alone [34]. Conversely, the benefit of ICI treatment was minimal in SCLC with liver metastasis compared with that in SCLC without liver metastasis [35]. The liver has unique immune tolerance characteristics; thus, an immunosuppressive microenvironment is formed during liver tumor metastasis. This limits the efficacy of immunotherapy [35]. García‐Mulero et al. [36] found that liver metastases had less infiltration of cytotoxic T lymphocytes (CTLs) than did lung, bone, and brain metastases. Yu et al. [37] demonstrated that liver metastases caused the disappearance of tumor‐specific CD8+ T cells throughout the body in a preclinical model. This is because of the apoptosis of activated T cells through their interaction with macrophages, which reduces the efficacy of immunotherapy. The unique immunological characteristics of liver metastases may reduce the benefit of ICIs on OS in SCLC. In this study, 15.2% of patients showed liver metastasis at the time of recurrence; this proportion was lower than that of patients with liver metastases enrolled in clinical trials. Patients who received 3D‐CRT were more likely to have undergone elective nodal irradiation (ENI), and those who underwent ENI tended to have a better prognosis than did those who received involved‐field radiotherapy. However, owing to the small number of cases, it is not possible to be certain.

The main AEs in this study were hematological toxicities, such as leukopenia, neutropenia, and thrombocytopenia, which occurred frequently. In the IMpower133 [16] and CASPIAN [17] trials, grade 3 or higher leukopenia occurred in 8.1% and 8%, neutropenia in 36.8% and 30%, and thrombocytopenia in 13.6% and 8% of patients, respectively. Myelosuppression was the reason for reducing the dose of platinum and etoposide in 20 (30.3%) patients; however, it was not the reason for treatment discontinuation, which was a manageable AE. Non‐hematological and gastrointestinal toxicities were relatively mild. Furthermore, there was no increase in the frequency of irAEs, such as interstitial lung disease, and none of the deaths were caused by irAEs. Regarding the risk of developing lung toxicity associated with CRT and ICI, studies on advanced non‐SCLC have shown that the incidence of lung toxicity increases in patients who received thoracic radiotherapy before ICI administration [38, 39, 40]. However, in the case of LD‐SCLC, although it occurs frequently, the details remain largely unknown. In this study, the incidence rate of pneumonia was 7.6%, and that of grade 3 or higher was 1.5%, suggesting that the treatment was well tolerated.

A phase III randomized ADRIATIC trial [41] showed that durvalumab consolidation after concurrent CRT significantly improved OS. Therefore, the combination of radiotherapy and immunotherapy is a promising approach for the treatment of intractable malignant tumors, such as SCLC.

Steel et al. [42] presented a mechanism by which a combination of drugs and radiation modulates the tumor microenvironment to produce a synergistic effect and improve prognosis [43]. Radiotherapy may simultaneously enhance immunostimulatory and immunosuppressive effects at the tumor site [43, 44]. Following radiotherapy, cytoplasmic DNA resulting from radiation‐induced double‐stranded DNA breaks activates cyclic GMP‐AMP synthase/stimulator of interferon genes (STING). In the STING pathway, type I interferon is released for anti‐presentation and immune stimulation [43, 45]. Moreover, radiation induces tumor apoptosis via cytotoxic T cells [46]. The concurrent use of radiotherapy and anti‐CTLA4 therapy may cause the upregulation of PD‐L1 expression, which is associated with T‐cell exhaustion and a decrease in the CD8/Treg ratio [47]. These changes promote immunosuppression and resistance to tumor treatment [47].

Therefore, maintenance therapy with ICIs after CRT is expected to become an innovative treatment option. Our analysis was conducted before the introduction of ICIs for LD‐SCLC, and this interpretation may change in the future. However, the efficacy of ICI retreatment following the ADRIATIC regimen requires further investigation.

This study has several limitations. First, the treatment effect may have been overestimated because of survivor bias and selection of patients who were suitable for platinum‐based chemotherapy with PD‐1 blockade at the time of study participation. Second, the retrospective study design lacked clear pre‐planned criteria for defining treatment effects and feasibility, limiting the interpretation of the results. The results relied on the subjective assessment of doctors, and there might have been variations in the treatment efficacy, survival data, and AEs. In the multivariable analysis, unmeasured confounding factors and patient selection bias might have influenced the results. Third, the sample size was small, with no power calculation or robust statistical hypothesis, introducing the possibility of bias in the results. Fourth, a control group was lacking, and it was difficult to evaluate the true effect of the treatment. Fifth, the details of anticancer treatment were determined at the discretion of the attending physician; therefore, the treatment content might have changed, or the treatment might have been delayed. To reduce this bias, we enrolled consecutive patients treated at participating facilities and performed a detailed analysis.

In conclusion, our real‐world data provide evidence that platinum, etoposide, and ICI combination chemotherapy may be a feasible treatment strategy with favorable efficacy in patients with recurrent disease after CRT for LD‐SCLC. These findings may provide a new direction for the pharmacological management of patients with recurrent LD‐SCLC. In the future, it is necessary to conduct prospective studies on the effects of platinum, etoposide, and ICIs in patients with recurrent LD‐SCLC.

## Author Contributions

All the authors have read and approved the final manuscript. Conceptualization and methodology: A.S. and H.I. Formal analysis and data curation: A.S., H.I., and K.K. Project administration, visualization, and writing – original draft preparation: A.S., T.A., and H.I. Supervision: K.K. and H.K. Investigation and resources: A.S., Y.S., K.Y., H.W., Y.T.‐K., A.T., T.O., N.Y., S.T., T.S., K.Y., Y.M., S.E., K.S., S.T., Y.M., Y.N., J.N., and T.T. Writing – review and editing: all authors.

## Conflicts of Interest

The authors declare no conflicts of interest.

## Supporting information


**Figure S1.** Kaplan–Meier curves showing OS of patients with limited disease small cell lung cancer who received CRT (median, 34.6 months). CI, confidence interval; CRT, chemoradiotherapy; OS, overall survival.

**Data S1** Tables.

## Data Availability

Data relevant to this study may be requested from the corresponding author but are not accessible to the public owing to ethical or privacy constraints.
