# Supplementary figures and images for: Significance of Platinum‐Based Chemotherapy With Programmed Death‐1 Blockade in Limited Disease Small Cell Lung Cancer: A Retrospective Study
Source: Thorac Cancer. 2025 Jun 30;16(13):e70118. doi: 10.1111/1759-7714.70118 (PMC12207248; doi:10.1111/1759-7714.70118)

## Slide 1
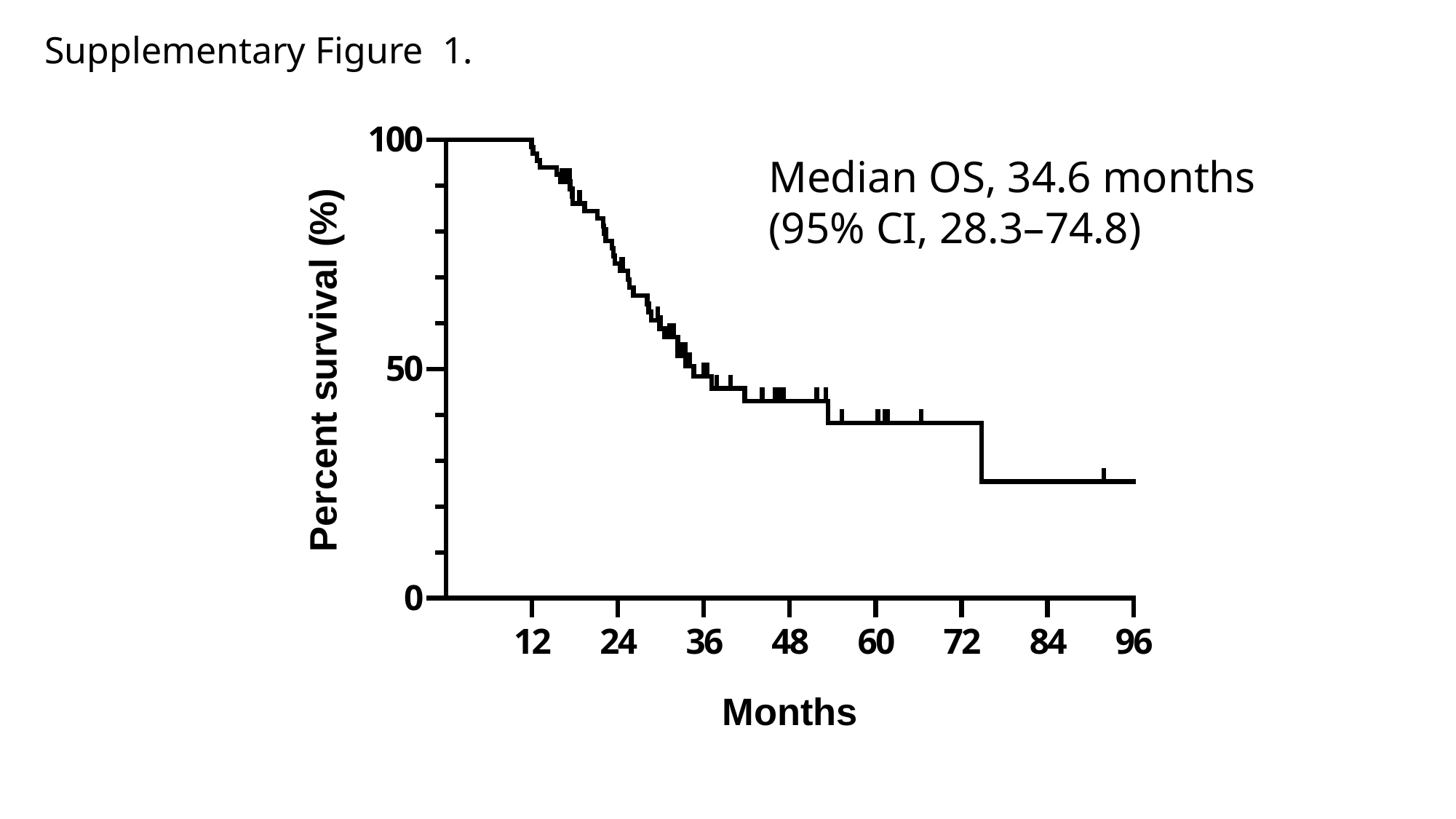

Supplementary Figure 1.
Median OS, 34.6 months
(95% CI, 28.3–74.8)

Supplement: Supplementary file 1 — Figure S1. Kaplan–Meier curves showing OS of patients with limited disease small cell lung cancer who received CRT (median, 34.6 months). CI, confidence interval; CRT, chemoradiotherapy; OS, overall survival. [file TCA-16-e70118-s001.pptx]
