# Supplementary material for: Significance of Platinum‐Based Chemotherapy With Programmed Death‐1 Blockade in Limited Disease Small Cell Lung Cancer: A Retrospective Study
Source: Thorac Cancer. 2025 Jun 30;16(13):e70118. doi: 10.1111/1759-7714.70118 (PMC12207248; doi:10.1111/1759-7714.70118)
Supplement: Supplementary file 2 — Data S1 Tables. [file TCA-16-e70118-s002.docx]

Supplementary Table 1. Patient characteristics at the time of chemoradiotherapy

|  | Total patients (N = 66) | % |
| --- | --- | --- |
| Age (years) |  |  |
| Median | 68 |  |
| Range | 52–81 |  |
| ECOG-PS |  |  |
| 0 | 38 | 57.6 |
| 1 | 27 | 40.9 |
| 2 | 1 | 1.5 |
| Disease stage |  |  |
| I | 0 | 0.0 |
| II | 8 | 12.1 |
| III | 58 | 87.9 |
| Radiotherapy fractionation method |  |  |
| Conventional | 8 | 12.1 |
| Accelerated hyperfractionated radiotherapy | 57 | 86.4 |
| Other | 1 | 1.5 |
| Radiation irradiation method |  |  |
| Three-dimensional conformal radiation therapy | 50 | 75.8 |
| Intensity-modulated radiation therapy | 16 | 24.2 |
| Irradiation area |  |  |
| Involved field irradiation | 26 | 39.4 |
| Elective nodal irradiation | 40 | 60.6 |
| Timing of radiotherapy in chemoradiotherapy |  |  |
| Concurrent | 59 | 89.4 |
| Sequential | 7 | 10.6 |
| Chemotherapy regimen |  |  |
| CDDP + etoposide | 45 | 68.2 |
| CBDCA + etoposide | 21 | 31.8 |
| Prophylactic cranial irradiation |  |  |
| Yes | 32 | 48.5 |
| No | 34 | 51.5 |

ECOG-PS, Eastern Cooperative Oncology Group Performance Status; CRT, chemoradiotherapy; CBDCA, carboplatin; CDDP, cisplatin

Supplementary Table 2. Univariate and multivariate analyses of progression-free and overall survival

| Variables | Median PFS  (months) | Univariate analysis | | | Multivariate analysis | | | Median OS  (months) | Univariate analysis | | | Multivariate analysis | | |
| --- | --- | --- | --- | --- | --- | --- | --- | --- | --- | --- | --- | --- | --- | --- |
|  |  | HR | 95% CI | *p*-value | HR | 95% CI | *p*-value |  | HR | 95% CI | *p*-value | HR | 95% CI | *p*-value |
| Sex |  |  |  |  |  |  |  |  |  |  |  |  |  |  |
| Male/female | 7.2/4.7 | 0.34 | 0.16–0.70 | **0.003** | 0.49 | 0.22–1.09 | 0.083 | 26.7/18.8 | 0.49 | 0.21–1.11 | 0.088 |  |  |  |
| Age |  |  |  |  |  |  |  |  |  |  |  |  |  |  |
| <75/≥75 | 5.8/6.0 | 0.89 | 0.46–1.70 | 0.728 |  |  |  | 26.7/16.3 | 0.48 | 0.23–1.03 | 0.062 |  |  |  |
| Performance Status (PS) |  |  |  |  |  |  |  |  |  |  |  |  |  |  |
| 0–1/2–3 | 6.0/3.7 | 0.38 | 0.13–1.09 | 0.072 |  |  |  | 25.0/15.3 | 0.34 | 0.10–1.14 | 0.08 |  |  |  |
| Recurrence at 12 months after CRT starting |  |  |  |  |  |  |  |  |  |  |  |  |  |  |
| Long/short | 9.3/5.2 | 0.55 | 0.31–0.99 | **0.049** | 0.53 | 0.28–0.97 | **0.042** | NR/16.8 | 0.24 | 0.09–0.60 | **0.002** | 0.29 | 0.11–0.79 | **0.015** |
| Presence of local recurrence at recurrence |  |  |  |  |  |  |  |  |  |  |  |  |  |  |
| Yes/no | 6.8/5.4 | 0.65 | 0.37–1.14 | 0.136 |  |  |  | 26.0/20.8 | 0.62 | 0.30–1.26 | 0.188 |  |  |  |
| Intracranial metastases at recurrence |  |  |  |  |  |  |  |  |  |  |  |  |  |  |
| Yes/no | 5.9/5.9 | 1.25 | 0.72–2.16 | 0.42 |  |  |  | 16.8/NR | 3.16 | 1.52–6.58 | **0.002** | 2.85 | 1.25–6.47 | **0.012** |
| Liver metastases at recurrence |  |  |  |  |  |  |  |  |  |  |  |  |  |  |
| Yes/no | 3.8/6.3 | 2.84 | 1.40–5.78 | **0.003** | 2.8 | 1.32–5.93 | **0.006** | 12.8/26.0 | 4.31 | 1.75–10.63 | **0.001** | 4.02 | 1.64–9.86 | **0.002** |
| Bone metastases at recurrence |  |  |  |  |  |  |  |  |  |  |  |  |  |  |
| Yes/no | 4.7/6.1 | 2.11 | 0.88–5.03 | 0.09 |  |  |  | 18.8/25.0 | 1.66 | 0.49–5.59 | 0.406 |  |  |  |
| Timing of radiotherapy in chemoradiotherapy |  |  |  |  |  |  |  |  |  |  |  |  |  |  |
| Concurrent/sequential | 5.9/5.4 | 1.13 | 0.45–2.85 | 0.788 |  |  |  | 24.9/NR | 0.75 | 0.22–2.50 | 0.643 |  |  |  |
| Radiotherapy fractionation method |  |  |  |  |  |  |  |  |  |  |  |  |  |  |
| Conventional/AHF | 5.5/5.9 | 1.02 | 0.43–2.41 | 0.946 |  |  |  | 12.2/25.0 | 1.82 | 0.70–4.74 | 0.214 |  |  |  |
| Radiation irradiation method |  |  |  |  |  |  |  |  |  |  |  |  |  |  |
| 3D-CRT/IMRT | 6.0/4.6 | 0.6 | 0.32–1.09 | 0.09 |  |  |  | 26.7/13.6 | 0.43 | 0.20–0.89 | **0.023** | 0.45 | 0.20–0.97 | **0.044** |
| Platinum agent at CRT |  |  |  |  |  |  |  |  |  |  |  |  |  |  |
| CDDP/CBDCA | 6.1/5.4 | 0.64 | 0.36–1.14 | 0.137 |  |  |  | 26.7/16.8 | 0.58 | 0.28–1.18 | 0.135 |  |  |  |
| Prophylactic cranial irradiation |  |  |  |  |  |  |  |  |  |  |  |  |  |  |
| Yes/no | 5.5/6.3 | 0.78 | 0.45–1.35 | 0.377 |  |  |  | NR/18.3 | 0.44 | 0.21–0.92 | **0.029** | 1.14 | 0.48–2.71 | 0.752 |
| NSE |  |  |  |  |  |  |  |  |  |  |  |  |  |  |
| High (≥16.4)/low (<16.4) | 5.0/5.6 | 0.82 | 0.43–1.58 | 0.566 |  |  |  | 26.0/25.0 | 0.92 | 0.36–1.87 | 0.652 |  |  |  |
| ProGRP |  |  |  |  |  |  |  |  |  |  |  |  |  |  |
| High (≥82)/low (<82) | 6.3/4.2 | 0.59 | 0.33–1.03 | 0.065 |  |  |  | 25.0/26.7 | 0.99 | 0.47–2.08 | 0.993 |  |  |  |
| BMI (kg/m^2^) |  |  |  |  |  |  |  |  |  |  |  |  |  |  |
| High (≥22)/low (<22) | 7.2/4.5 | 0.42 | 0.24–0.75 | **0.003** | 0.57 | 0.30–1.08 | 0.087 | 26.0/18.8 | 0.75 | 0.36–1.57 | 0.454 |  |  |  |
| NLR |  |  |  |  |  |  |  |  |  |  |  |  |  |  |
| Low (<5)/high (≥5) | 5.9/5.9 | 0.95 | 0.51–1.79 | 0.89 |  |  |  | 24.9/NR | 0.94 | 0.41–2.17 | 0.897 |  |  |  |
| PLR |  |  |  |  |  |  |  |  |  |  |  |  |  |  |
| Low (<185)/high (≥185) | 6.2/5.3 | 1.88 | 1.09–3.24 | **0.022** | 0.87 | 0.47–1.62 | 0.678 | 24.9/18.3 | 0.9 | 0.46–1.78 | 0.779 |  |  |  |
| ALI |  |  |  |  |  |  |  |  |  |  |  |  |  |  |
| Low (<24)/high (≥24) | 5.4/6.1 | 1.21 | 0.70–2.07 | 0.488 |  |  |  | 18.8/26.0 | 1.18 | 0.60–2.32 | 0.626 |  |  |  |
| PNI |  |  |  |  |  |  |  |  |  |  |  |  |  |  |
| Low (<45)/high (≥45) | 4.9/7.2 | 1.88 | 1.09–3.24 | **0.022** | 1.33 | 0.71–2.50 | 0.365 | 15.9/26.7 | 1.8 | 0.90–3.58 | 0.092 |  |  |  |

Bold font indicates statistically significant differences.

PFS, progression-free survival; OS, overall survival; HR, hazard ratio; CI, confidence interval; PS, performance status; AHF, accelerated hyperfractionated radiotherapy; 3D-CRT, three-dimensional conformal radiation therapy; IMRT, intensity-modulated radiation therapy; CDDP, cisplatin; CBDCA, carboplatin; NSE, neuron-specific enolase; proGRP, pro-gastrin-releasing peptide; BMI, body mass index; GPS, Glasgow prognostic score; NLR, neutrophil-to-lymphocyte ratio; PLR, platelet-to-lymphocyte ratio; ALI, Advanced Lung Cancer Inflammation Index; PNI, prognostic nutritional index
